# Cesœrean Operation

**Published:** 1851-07

**Authors:** M. M. Rodgers

**Affiliations:** Paris


### ART. VII.—*Cesærean Operation.*

*Paris, January* 20, 1851.

Mr. Editor: *Dear Sir*,—I send you for publication an account of a case of Cæsarean operation, which I have just seen performed by M. Paul Dubois, in the Hospital "Clinique d' Accouchments." This operation, although far more common than in the United States, is by no means of frequent occurrence in Europe. M. Dubois, if I understood him correctly, said he had made the section eight times before.

The subject of the operation was an in-patient of the hospital; single woman, 24 years of age, primiparous, dwarfish, of rachitic constitution, nervo-bilious and lymphatic temperament, with deformed pelvis and inferior extremities. The pelvis was compressed so as to leave only 1¼ inch in the antero-posterior diameter, which was insufficient for the delivery of the child even after embryotomy. Labor commenced at the full period of gestation, and had been progressing slowly for about six hours, the amniotic fluid having been discharged during that time. Difficulty being apprehended by the "internes" and "chef. du clinique" in attendance, M. Dubois, Physician Accoucheur, of this hospital, was called in: after examination per vaginam, the Professor, by the concurrent advice of Prof. De Paul, decided upon the necessity of the section. This was at 9 o'clock in the evening, the woman being then somewhat exhausted, and the child still living, as shown by auscultation: the operation was, however, deferred till the next day at 10 o'clock. The patient was brought into the amphitheatre somewhat more feeble than the night before, although under the effect of anodynes and stimulants: she was laid upon her back on the operating bed,.with the thighs flexed upon the body, and the shoulders raised.

The operation was commenced, (without chloroform,) by making an incision just opposite the umbilicus, and extending to the symphysis pubis, about eight inches: the first incision was made through the integuments; a small opening was then made through the peritoneum, and the incision finished by a bistoury and grooved director: the next incision was made through the walls of the uterus, about six inches long, when the child appeared in sight; it was extracted by the feet, dead; the cord was tied and the placenta extracted by the same orifice. The operation occupied about eight minutes exclusive of dressing. The bleeding was only slight from the incision; the edges of the wound in the abdominal walls was brought into coaptation and secured by interrupted quilled sutures, the incision in the uterus being perfectly closed by its contraction; adhesive straps, charpie, a compress and bandage around the body, finished the dressing. The patient, who suffered much from pain, and was much exhausted, was removed to her ward, and allowed an anodyne and hot wine and water: she, however' was unable to rest, and reaction not taking place, she sunk rapidly, and died of collapse, thirty-six hours after the operation.

This was, doubtless, a fair case for the operation, and offered the only hope of saving either the mother or child: but the time to save the child was while it was alive; and after that was dead, the mother was too much exhausted to leave much hope of her recovery from so severe an operation; so that the delay in the case certainly was the cause of losing one if not both lives. But as I intended only to give the details of the operation, which was skilfully performed, I shall give no opinions, but leave others to draw such conclusions, and make such reflections as they please.

Yours respectfully,
M. M. RODGERS, M. D.

